# Married

**Published:** 1867-07

**Authors:** 


***Married.***

At Galva, Ill., June 3d, 1867, by Rev. L. D. Gowen, Dr. T. NEWTON BOOE, of Loda, Ill., and MISS EFFIE L. BURNETTE, of Galva.

At Jonesboro, Ill., April 23d, 1867, by Rev. E. B. Olmstead, of Caledonia, GEO. W. SCHUCHARD, M.D., formerly of Golconda, Ill., to Miss HELEN A. DOUGHERTY, of this place.

April 17th, 1867, at the residence of the bride's father, in White Cloud, Iowa, by Rev. Dr. J. H. Wing, JAMES L. GANDY, M.D. formerly Ass't.-Surg., U.S.A. and Miss MARY E. OTT. The former a son of Dr. O. Gandy of Ind., The latter a daughter of Hon. George Ott, of White Cloud, Iowa.

